# A comparative study of organoid-derived and cell line-derived intestinal epithelial models

**DOI:** 10.1371/journal.pone.0346560

**Published:** 2026-04-16

**Authors:** Melis Asal, Nanne J. Paauw, Hetty J. Bontkes, Sandra J. van Vliet, Reina E. Mebius, Susan Gibbs

**Affiliations:** 1 Department of Molecular Cell Biology and Immunology, Amsterdam UMC, Vrije Universiteit, Amsterdam, The Netherlands; 2 Amsterdam Institute for Immunology and Infectious diseases, Amsterdam, The Netherlands; 3 Department of Laboratory Medicine, Laboratory Specialized Diagnostics and Research, Section Medical Immunology, Amsterdam University Medical Center, Amsterdam, The Netherlands; 4 Cancer Center Amsterdam, Amsterdam, The Netherlands; 5 Department of Oral Cell Biology, Academic Centre for Dentistry Amsterdam (ACTA), University of Amsterdam and Vrije Universiteit, Amsterdam, The Netherlands; Macau University of Science and Technology, HONG KONG

## Abstract

**Background:**

In vitro intestinal epithelial models are being developed for studying gut physiology, barrier function, and drug transport. There are two major in vitro models which are currently used: cell line-derived monolayers (such as Caco-2/HT29 cocultures) and primary cell monolayers derived from human intestinal organoids.

**Aim:**

To conduct a side by side comparison of organoid-derived and cell line-derived intestinal epithelial monolayers, assessing their morphological features, barrier performance, and secretome under similar culture conditions.

**Methods:**

Human duodenum-derived organoids and Caco-2/HT29 cocultures (9:1 ratio) were seeded onto Transwell inserts and were harvested at the same time for comparative analysis. Barrier function was assessed by transepithelial electrical resistance (TEER) and Lucifer Yellow permeability. Structural characterization was performed using immunofluorescence microscopy, scanning and transmission electron microscopy. Secretion of growth factors and chemokines was quantified on apical and basal sides via multiplex assays.

**Results:**

Both models formed intact epithelial barriers with robust tight junctions and displayed appropriate apical-basolateral polarization, confirmed by the presence of microvilli. However, the organoid-derived model exhibited occasional villus-like protrusions and ultrastructural crypt-like invaginations, homogenous secretion of Mucin 2 over the surface, and release of epithelial growth factor whereas the cell line model exhibited longer and more densely packed microvilli and higher apical secretion of vascular endothelial growth factor. The barrier properties were comparable except that the organoid model had decreased permeability (Lucifer Yellow).

**Conclusions:**

This study highlights key structural, functional, and molecular similarities and differences that influence the physiological relevance and application scope of each model. It enables educated decision making when choosing the best model for a particular “Context of Use” weighing the pros and cons between experimental convenience and physiological relevance.

## Introduction

The native small intestinal epithelium is a highly specialized, dynamic tissue whose hallmark villus-crypt architecture and heterogeneous cellular composition together amplify the absorptive surface area, strengthen barrier function, and provide structural and functional cues. The epithelial layer consists of multiple differentiated cell types, primarily absorptive enterocytes, which form dense apical microvilli to facilitate nutrient uptake. Goblet cells interspersed among enterocytes secrete mucus, predominantly composed of Mucin 2 (MUC2), which protects the epithelium and maintains intestinal homeostasis. Additionally, enteroendocrine cells produce hormones that regulate digestion and gut physiology, while crypts house stem and progenitor cells responsible for continuous epithelial renewal [1–3].

Tight junction proteins, such as Zonula occludens (ZO-1), form critical barriers between epithelial cells, regulating paracellular permeability and maintaining selective barrier integrity [4]. Notably, barrier properties including tight junction composition and permeability vary along the intestinal tract. In vivo measurements using Ussing chambers reported in the literature show that intestinal permeability decreases along the proximal-to-distal axis, with the duodenum being more permeable than the jejunum and colon [5]. These differing transepithelial electrical resistance (TEER) values along the intestine have evolved to optimally accommodate location-dependent nutrient absorption and defense [5–7].

Intestinal epithelial models are indispensable tools for studying gut physiology, barrier function, drug absorption and host-microbe interactions in vitro. Two main approaches dominate the field: immortalized intestinal cell lines derived from colorectal adenocarcinoma such as Caco-2 and HT29 which represent enterocytes and goblet cells respectively, and primary cell-derived human intestinal organoids. Each model offers distinct advantages and limitations depending on the research question and application. Cell line-based models (Caco-2 and HT29) are widely used due to their ease of culture, scalability, reproducibility, and therefore suitability for high-throughput screening. These cells readily form polarized monolayers and are routinely used to model human small intestine for evaluating permeability, drug transport, and toxicological responses [8–12]. However, their cancerous origin and long-term passaging can result in phenotypic drift and limited physiological relevance [13]. In contrast, cystic organoids originate from adult stem cells and better recapitulate the cellular heterogeneity and structural features of native intestinal tissue such as differentiated cell lineages (enterocytes, goblet cells, Paneth cells and enteroendocrine cells), polarized epithelium with mature tight junctions and apical microvilli, active mucus secretion, physiologic rates of cell proliferation and turnover, and even self‑organized crypt‑like and villus‑like domains that recapitulate key aspects of in vivo regionalization. When disrupted and grown as monolayers, these organoid-derived models retain the ability to differentiate into multiple epithelial lineages, maintaining their complex morphology and functional polarization [14–22]. Despite their biological advantages, organoid-based systems are technically more demanding, require access to primary tissue, and introduce donor-dependent variability [23]. Furthermore, current in vitro cell line and organoid-derived monolayer models generally have a less leaky barrier function and higher TEER value to that observed in vivo [5,24].

The aim of this study was to perform a direct comparison between the two monolayer intestine epithelium culture models grown on Transwell membranes. A coculture model of Caco-2 and HT29 cells and a human small intestine (duodenum) organoid-derived monolayer were cultured in parallel with time matched end point analysis with regards to polarization, microvilli density and crypt formation (electron microscopy); barrier integrity (TEER and paracellular permeability) and secretome of homeostatic (EGF) and angiogenic (VEGF) growth factors as well as chemokines from the apical and basolateral sides of the models.

Our results enable educated decision making when choosing the best model for a particular “Context of Use” weighing the pros and cons between experimental convenience and physiological relevance.

## Materials and methods

### Tissue specimens

Human duodenal tissues were obtained as residual material from Whipple procedures through the Amsterdam University Medical Center biobank. All tissue collection and use were conducted in accordance with the ‘Code of Conduct for Health Research’ established by COREON (Commissie Regelgeving Onderzoek), with written informed consent from patients and approval from the local medical ethics committee of the Amsterdam University Medical Center.

### Cell isolation and culture

For generation of primary small intestine organoids, crypts were isolated from human duodenum as previously described [25,26]. Following a 30-minute incubation on ice in EDTA chelation buffer, tissue samples were processed to release epithelial crypts, which were then embedded in growth factor-reduced (GFR) Matrigel® (Corning, Corning, NY, USA) and plated as 25 μl droplets in 24-well plates. Organoids were cultured in IntestiCult™ Organoid Growth Medium (OGM; StemCell Technologies, Vancouver, Canada) at 37°C and 5% CO₂, with medium refreshed every other day and weekly passaging. Before seeding onto Transwell inserts, organoids were dissociated by breaking down the Matrigel® domes using Gentle Cell Dissociation Reagent (StemCell Technologies) and filtering the suspension through 40 μm strainers to obtain a single cell suspension.

The C2BBe1 cell line, a clone of human colon adenocarcinoma Caco-2 cells (CRL-2102, ATCC, Middlesex, UK), was maintained in DMEM supplemented with 0.01 mg/ml human transferrin (Sigma-Aldrich, St. Louis, MO, USA), 10% fetal bovine serum (FBS; Corning), and 1% penicillin-streptomycin (P/S). HT29 cells (HTB-38, ATCC) were cultured in McCoy’s 5a Medium (Thermo Fisher Scientific, Waltham, MA, USA), also supplemented with 10% FBS and 1% P/S. Both cell lines were cultured at 37°C and 5% CO₂ and were used for experiments upon reaching 90% confluency.

Caco-2 and HT29 cells were obtained from ATCC at passages 47 and 130, respectively, and used for experiments between passages 48–50 (Caco-2) and 131–133 (HT29). Cells were routinely monitored for morphology and growth characteristics.

### Construction of the models

**Fig 1:**
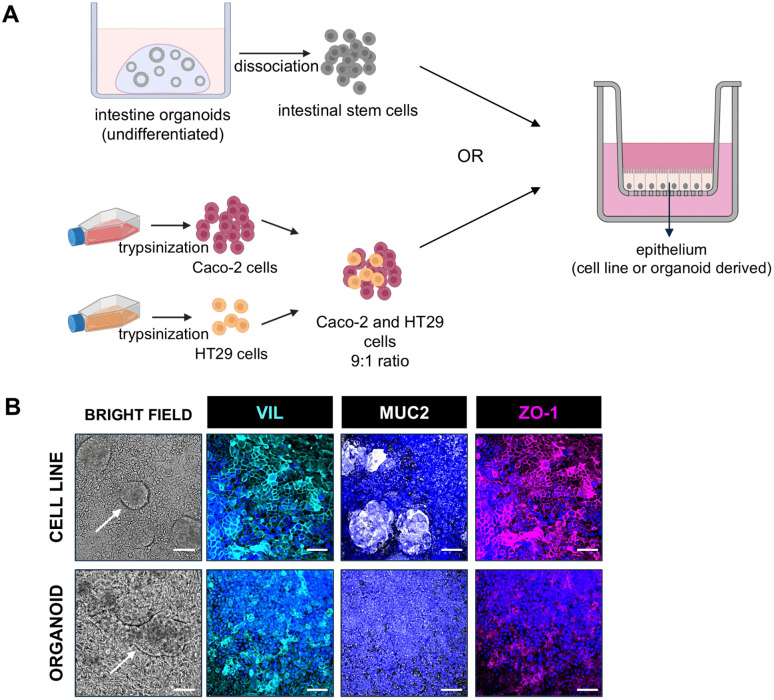
Characteristics of in vitro intestinal epithelium models. a) Schematic illustration of the construction of intestinal epithelial monolayer Transwell culture systems using either organoid cells or cell lines. Created in BioRender. b) Characterization of epithelial monolayers growing on surface of Transwell membrane. Bright field images: white arrow indicates cell clusters in cell line model and protrusions on the surface of organoid-derived model. Immunofluorescent images: VIL (enterocyte apical brush border): cyan; MUC2 (goblet cell mucin): white; ZO-1 (tight junctions): magenta. Data representative of n = 3 independent experiments each with 2 intra-experimental replicates. Scale bar: 50 μm.

Single cell suspensions obtained from either trypsinized Caco-2 and HT29 cells (9:1 cell number ratio, cell line model) or primary human duodenal epithelial organoids (passage 3, organoid model) were passed through a 40 µm cell strainer to remove aggregates, counted using an automated cell viability counter (Cellometer Auto 2000, Nexcelom Bioscience, Lawrence, MA, USA) and seeded at a density of 250 × 10³ cells per insert onto 12 mm Transwells (Corning) with 0.4 μm pore size, pre-coated with GFR Matrigel®. Cell line models were initiated 7 days prior to organoid-derived models so that both models were harvested on the same day. Cell line models were maintained in Caco-2 medium, while organoid models were cultured in IntestiCult™ OGM for the first 7 days, followed by IntestiCult™ Organoid Differentiation Medium (ODM; StemCell Technologies) for the final 7 days, applied to both apical and basal compartments. A schematic of the models is illustrated in Fig 1a.

### Measurement of TEER

TEER was assessed at the end of the culture period using a Millicell ERS-2 Voltohmmeter (MERS00002, Merck Millipore, MA, USA), following the manufacturer’s protocol. Briefly, the disinfected electrode was rinsed with culture medium and positioned vertically, with the shorter tip placed in the apical compartment and the longer tip in the basal compartment. Inserts without cells, maintained under identical conditions, were used as blanks. Unit area resistance (UAR, Ωcm²) was calculated as follows:


UAR=(SampleResistance−BlankResistance)xMembraneSurfaceArea

where *Sample Resistance* refers to the electrical resistance measured across cell-seeded Transwell inserts, *Blank Resistance* is the resistance of cell-free inserts cultured under identical conditions using the same medium, and *Membrane Surface Area* corresponds to the surface area of the Transwell membrane covered by the cell layer.

### Barrier integrity assessment using lucifer yellow

To assess epithelial barrier integrity, both the apical and basal compartments of the Transwell system were gently rinsed with HBSS buffer. 0.1 mg/ml Lucifer Yellow (Sigma-Aldrich) was added to the apical side (200 μl), and 700 μl of HBSS was added to the basal side. Samples were incubated for 1 hour at 37 °C, protected from light. After incubation, 200 μl was collected from the basal compartment and transferred to a 96-well plate. Fluorescence was measured using a fluorometer (excitation: 485 nm; emission: 527 nm). Permeability was expressed as % of apically applied Lucifer Yellow detected in the basolateral compartment (referred to as % permeability).

Permeability was calculated using the following formula:


$$\%Permeability=\frac{LuciferYellow-HBSS}{Sample-HBSS}x\text{1}\text{0}\text{0}$$

where *Sample* is the measured fluorescence intensity of the basal compartment, *HBSS* is the background signal, and *Lucifer Yellow* is the fluorescence intensity of a 0.1 mg/ml Lucifer Yellow solution.

### Bright field imaging

Bright-field images were acquired using the EVOS™ XL Core Imaging System (Thermo Fisher Scientific) equipped with a 10 × objective (NA 0.25).

### Immunofluorescence staining

The semipermeable membrane was cut off the Transwell insert according to the manufacturer’s guidelines (Preparation of Transwell Inserts for Histology Guideline for Use, Corning) and washed with PBS. After fixation with 4% paraformaldehyde, samples were washed again and stored at 4°C until staining. For staining, samples were incubated with 1% BSA (w/v) and 0.3% Triton X-100 (v/v) in PBS for 1 hour at RT, followed by overnight incubation at 4°C with primary antibody (anti-Villin-1:100 (MA5−12227, Thermo Fisher Scientific), anti-Mucin 2–1:200 (NBP2−66961, Novus) or anti-Zonula occludens1–1:100 (Bs-1329r, Bioss Antibodies)) diluted in 2% normal goat serum in PBS. After washing 3x with PBS, secondary antibody ((Alexa Fluor® 488 goat anti-mouse IgG-1:100 (A11029, Molecular Probes, Invitrogen) or Alexa Fluor® 555 goat anti-rabbit IgG-1:100 (A21429, Molecular Probes, Invitrogen)) diluted in 2% goat serum was applied for 2 hours at RT. Samples were placed on a slide, mounted with Fluoroshield Mounting Medium with DAPI (Abcam, Cambridge, UK), and covered with a coverslip. Samples were imaged using VS200 slide scanner (Olympus, Tokyo, Japan). The images were analyzed using QuPath software (version 0.4.4) [27].

### Electron microscopy imaging

For scanning electron microscopy (SEM), samples were fixed in 2% paraformaldehyde and 2.5% glutaraldehyde in 0.1 M phosphate buffer, followed by dehydration through a graded ethanol series. After drying, the samples were mounted on metal stubs and coated with a thin layer of platinum/palladium via sputter coating. Imaging was performed using a Gemini 300 Sigma microscope (Zeiss, Oberkochen, Germany).

For transmission electron microscopy (TEM), samples were similarly fixed in 2% paraformaldehyde and 2.5% glutaraldehyde in 0.1 M phosphate buffer, post-fixed with 1% osmium tetroxide, dehydrated through increasing ethanol concentrations, and embedded in Epon resin. Ultrathin sections (70 nm) were cut using a Leica UM UC7 ultramicrotome and collected on copper grids. The sections were then stained with uranyl acetate and lead citrate before visualization on a Talos L120c TEM equipped with a BM-Ceta 16M camera (Thermo Fisher Scientific).

### Measurement of growth factor and chemokine secretion in culture supernatant

The LEGENDplex Human Growth Factor Panel (13-plex) for analytes ANG-2, EGF, EPO, FGF-basic, G-CSF, GM-CSF, HGF, M-CSF, PDGF-AA, PDGF-BB, SCF, TGF-β and VEGF and a LEGENDplex subpanel of proinflammatory chemokine panel 1 for analytes CCL20, CXCL1, CXCL8, CXCL10 and CXCL11 were used to assess chemokine and growth factor secretion, according to the manufacturer’s instructions (BioLegend, San Diego, California, U.S.). Samples were acquired on Attune NxT Flow Cytometer (Thermo Fisher Scientific). For secretome analysis, cultures were maintained in new medium for 24 h prior to supernatant collection. Culture supernatants were harvested and stored at −20 °C until analysis. As a background control, culture medium without cells was incubated for 24 h. Background values measured in medium-only controls were subtracted from cell supernatant values.

### Statistical analysis

All statistical analysis was performed using GraphPad Prism software (version 9.5.1) (GraphPad Software Inc., La Jolla, CA, USA). Student’s t-test was used to assess the differences. The differences were considered significant when p < 0.05. All values are reported as standard deviation; * = p < 0.05; ** = p < 0.01. All figures summarize data from n = 3 independent experiments with 2 intra-experimental replicates. A different duodenum donor was used for each organoid experiment.

## Results

### Comparison of structural characteristics of cell line and organoid-derived epithelial models

Epithelial monolayers derived from cell lines or organoids grown on Transwell inserts were compared for morphological and cell type specific (enterocyte and goblet cell) similarities and differences (Fig 1). Both epithelial models formed a confluent layer over the Transwell membrane. However, the cell line model showed clear circular clumping of cells whereas the organoid-derived model showed slight protruding villus-like structures. These protrusions were absent in cell line-based models, which appeared flat except for the observed clumps (Fig 1b, white arrows).

Immunofluorescence confirmed the presence of key epithelial markers in both models. Villin (VIL, cyan) which stains apical brush borders, was equally present over the surface of both models highlighting the presence of differentiated enterocytes. Goblet cells were identified by Mucin 2 (MUC2, white). MUC2 was notably more uniformly present over the organoid-derived model surface than in the cell line model, where the circular clumps in the latter were found to consist of the MUC2 positive HT29 cells. Tight junctions were defined by ZO-1 (magenta), outlining cell-cell boundaries in both models (Fig 1b).

**Fig 2:**
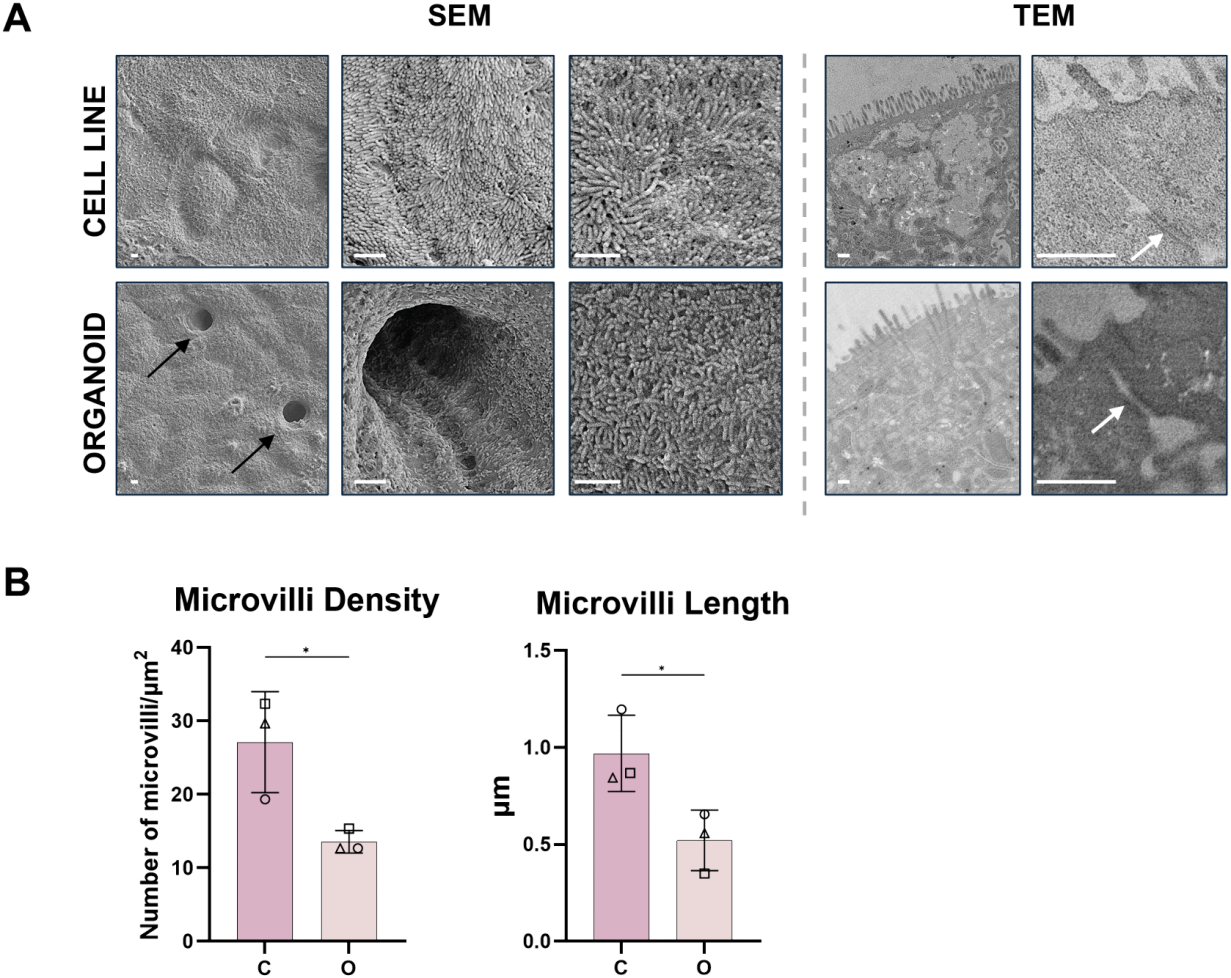
Ultrastructural assessment of epithelial models. **a)** Scanning electron microscopy (SEM) showing surface and transmission electron microscopy (TEM) showing tissue cross section images of the respective models. Microvilli are seen covering the surface of both models. Black arrows indicate invaginations resembling early crypts in organoid model. White arrows indicate tight junctions. Scale bar: 1 μm in all SEM images and 0.5 μm in all TEM images. Differences in the apparent size of the scale bars reflect differences in image magnification. **b)** Quantification of microvilli density and length in cell line model and organoid monolayer model. C: cell line model; O: organoid model. Data representative of n = 3 independent experiments. All values are reported as standard deviation; * = p < 0.05.

In order to identify similarities and differences at the surface and subcellular ultramicroscopic level, both models were analyzed with SEM and TEM (Fig 2a). Both models showed densely packed apical microvilli, characteristic of functional enterocytes. In the cell line model, microvilli density was significantly higher (~27 per μm^2^ vs ~ 13 per μm^2^) and the microvilli were significantly longer (~1 μm vs ~ 0.5 μm) than in the organoid model (Fig 2b).

Notably, in organoid-derived monolayers, microvilli-covered invaginations reminiscent of crypt-like structures were observed, which were absent in the cell line-derived model (Fig 2a, SEM, black arrows). TEM images confirmed the presence of tight junctions (Fig 2a, white arrows) between adjacent enterocytes in both models.

Taken together, these results indicate that both models possess structural epithelial characteristics, with the organoid-derived model exhibiting villus and crypt-like protrusions and a uniform mucus distribution.

### Comparison of barrier integrity

**Fig 3:**
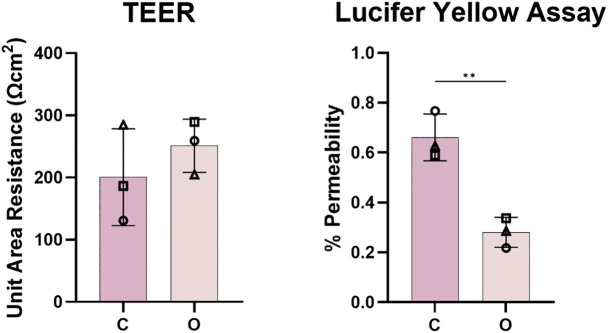
Evaluation of epithelial barrier properties with transepithelial electrical resistance (TEER) and paracellular permeability (Lucifer Yellow Assay) measurements. C: cell line model; O: organoid model. Data representative of n = 3 independent experiments each with 2 averaged intra-experimental replicates. All values are reported as standard deviation; ** = p < 0.01.

Barrier function was evaluated using TEER and Lucifer Yellow permeability assays (Fig 3). Both models developed TEER values (approximately 200 Ωcm²) reflecting the establishment of epithelial barriers, with organoids showing slightly higher resistance.

Notably, the Lucifer Yellow permeability assay revealed a distinct difference in paracellular permeability between the models. While both models restricted dye passage efficiently, the organoid-derived model exhibited significantly lower permeability, consistent with tighter junctional complexes (Fig 3).

### Secretion of soluble proteins by epithelial models

Secretion of growth factors and chemokines were next compared. In addition to comparing the secretome between the two models, the secretome was also compared between the apical and basolateral sides of the models. Values were background corrected to 24 hour medium only controls.

**Fig 4:**
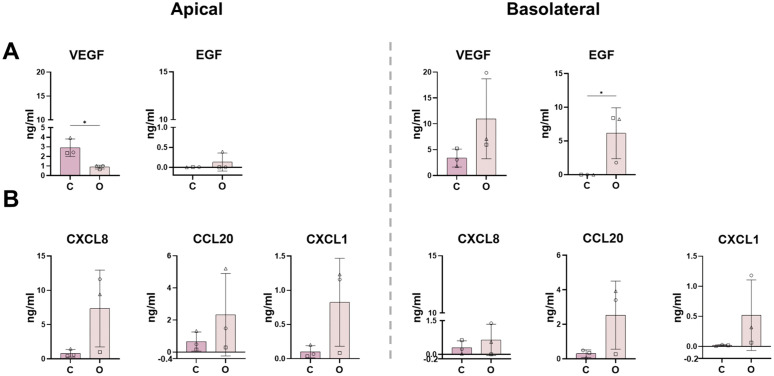
Soluble protein secretion from epithelial models into culture supernatant a) growth factor (VEGF and EGF) and b) chemokine (CXCL8, CCL20, CXCL1) secretion was detected using cytometric bead arrays. Apical and basal indicates analysis of culture medium collected from apical or basolateral compartments of the Transwell culture system, respectively. Values shown are background corrected to 24 hour medium only controls. C: cell line model; O: organoid model. Data is representative of n = 3 independent experiments with 2 averaged intra-experimental replicates. All values are reported as standard deviation; * = p < 0.05; ** = p < 0.01.

Growth factor analysis (Fig 4a) revealed model-specific secretion patterns. EGF, which functions as a mitogen that stimulates epithelial proliferation and repair [28], was secreted significantly in higher levels in the organoid-derived model, from the basolateral side, compared to the cell line-derived model. This basolateral bias is consistent with the predominantly basolateral localization of the EGF receptor in polarized intestinal epithelial cells [29,30]. Conversely, VEGF (promotes angiogenesis) [28,31], was secreted at significantly higher levels in the cell line-based model in the apical compartment. Apical enrichment of VEGF has been reported in epithelial systems and may reflect epithelial contributions to luminal or mucosal signaling environments [32,33]. Other studied growth factors (EPO, FGF-basic, G-CSF, GM-CSF, HGF, M-CSF, PDGF-AA, PDGF-BB, SCF and TGF-β) were detected but did not show any meaningful difference between models.

Chemokine secretion profiles (Fig 4b) showed that both models secreted CXCL8, CCL20, and CXCL1. Differences between the two models were not statistically significant but were generally higher in organoid cultures. Other studied chemokines (CXCL10 and CXCL11) were under the detection limit of the assay.

Collectively, these data highlight that while both models exhibit functional polarization regarding soluble protein release, the organoid-derived model demonstrates higher secretion of the major homeostatic factor EGF and inflammatory chemokines, whereas the cell line model displays higher apical secretion of angiogenic mediators. Notably, greater variability was observed in the organoid-derived model, likely reflecting inter-donor differences.

## Discussion

In this study, we present a direct comparison of two organotypic intestinal monolayer models composed of a cell line-derived or organoid-derived monolayer epithelium. While previous studies have assessed some features of Caco-2/HT29 versus organoid-derived models [14,18], our study directly compares structural and biochemical properties between the models using TEER and permeability, immunofluorescence for VIL, MUC2, ZO-1, SEM and TEM as well as secretome analysis thus providing a comprehensive side-by-side comparison of morphology and barrier-related physiology of the two models.

In our study, TEER measurements confirmed that both organoid-derived and cell line-based monolayers established intact epithelial barriers (TEER approx. 200 Ωcm²). Despite similar TEER, the organoid-derived duodenal monolayers showed ∼3-fold lower Lucifer Yellow permeability than the cell line coculture. Because TEER primarily reports small-ion conductance whereas Lucifer Yellow permeability reports rare, paracellular leaks, these two readouts do not always change in parallel to each other [34]. The data therefore indicate that the organoid monolayers possess fewer or smaller leak-pathway defects, consistent with more continuous tight-junction architecture, rather than simply higher overall ionic resistance. An additional factor which should be taken into account could be the mucus coverage. Histology revealed that mucus in cell line coculture was present only in patches associated with HT29 cells in contrast to the organoid-derived cultures where it exhibited a more uniform, although modest, mucus layer. The localized expression in the cell line coculture may reflect intracellular or membrane-bound MUC2 expression rather than extensive mucus secretion. Even limited mucus may slow paracellular dye passage, potentially contributing to the reduced Lucifer Yellow permeability observed in organoid monolayers [35].

HT29 cell clustering was observed consistently in our coculture model. Similar localized mucin expression patterns have also been reported in the literature for cocultures employing the same parental HT29 cell line (HTB-38), where mucus production was described as non-uniform and spatially restricted rather than evenly distributed [36]. This suggests that clustering and patchy organization occurs in HTB-38 based coculture systems. Such behavior may relate to the use of the parental HT29 (HTB-38) line rather the mucus-enriched subclone HT29-MTX which has been reported in Caco-2 cocultures to form relatively homogeneous epithelial layers [12,37]. This intrinsic phenotypic heterogeneity may be associated with the cancer origin of HT29 cells.

Throughout this study, analyses and interpretation are framed specifically within the context of the duodenum, reflecting the regional origin of the primary tissue used to generate the organoid-derived monolayers. Intestinal epithelial structure and function vary along the proximal–distal axis of the small intestine, and segment identity should therefore be considered when comparing in vitro models. Accordingly, the observations described here are interpreted in relation to duodenal epithelium rather than generalized to the entire small intestine. In native human duodenum, absorptive enterocytes display a dense microvillus brush border, with ultrastructural studies reporting long and numerous microvilli in duodenum and jejunum that decrease toward the ileum [3]. Although classic microvilli density values (~40–50 µm ⁻ ²) are often cited, these originate from rodent tissue and direct in vivo quantitative data for human duodenum is absent. However, it is known that in native duodenum, microvilli typically reach lengths of ∼1 µm and are densely packed [38,39]. In our study, the cell line co‑culture exhibited ∼1 µm long densely packed physiological microvilli while the organoid model fell short on the length and density of microvilli. Although it can not be ruled out that this difference is due to insufficient epithelial differentiation, it should be noted that the organoids used for constructing the model were derived from a 7-day differentiation protocol which is the standard in literature for constructing primary intestinal organoids [25,40]. Also, microvilli observed in human duodenal organoid-derived epithelial monolayers reported in the literature appear similar in density and length to those observed in our model [14]. Our finding may be due to differences in epithelial organization imposed by planar monolayer culture, which has been shown to alter tissue architecture and morphogen signaling despite preserving differentiation capacity. Future optimization of physical and biochemical parameters, such as substrate properties, shear stress, or trophic factor gradients, may further enhance brush border maturation in organoid-derived monolayers.

Our growth factor secretion analysis revealed that the cell line model secretes higher amounts of VEGF at the apical side while the organoid‑derived monolayers release significantly more EGF into the basolateral compartment compared to the Caco‑2/HT29 co‑culture. Given EGF’s role in driving epithelial proliferation, differentiation, and wound repair, this elevated EGF secretion suggests sustained barrier renewal under homeostatic conditions [29,30]. Both Caco-2 and HT29 cells originate from colorectal adenocarcinomas and, despite differentiation into enterocyte and goblet-like cells under certain conditions, they retain some features of transformed cells. One such feature is the sustained secretion of pro-angiogenic factors like VEGF, even in the absence of exogenous angiogenic stimuli [29]. This may result from persistent activation of signaling pathways (e.g., MAPK, PI3K/AKT, or HIF-1α pathways) that drive VEGF expression in cancer cells [32,41]. Although cancer cells in vivo promote angiogenesis through basolateral secretion of VEGF, no difference with apical secretion was observed in our cell line model. In contrast, organoid-derived epithelium secreted VEGF predominantly basolaterally, consistent with their physiological role in promoting subepithelial angiogenesis. Chemokine secretion (CXCL8, CCL20, CXCL1) was detectable in both models but did not differ significantly. However, a trend to higher chemokine release was clearly seen in the organoid model compared to the cell line model.

Our study expands on the results obtained by others. While previous studies have examined individual intestine epithelial model systems, only a few have conducted a direct, side-by-side functional and structural comparison using matched end time point analysis with extensive readouts comparing intestinal cell line derived and organoid-derived monolayer cultures. One study highlighted that organoid monolayers could include specialized cell types (enteroendocrine cells, M cells), which are absent in Caco-2 and HT29 models. This allows for more accurate modeling of particle uptake and immune responses in the gut [30]. Another study showed that organoid-derived monolayers have much higher activities of key drug-metabolizing enzymes (CYP3A4, CES2), better efflux transporter function (P-glycoprotein) and inducibility of CYP3A4 compared to Caco-2 cells. The gene expression profile of organoid monolayers is also much closer to that of adult human duodenum than Caco-2 cell based models [14]. More recently, Streekstra et al. (2024) directly compared organoid-derived and Caco-2 monolayers in bidirectional drug transport assays. Both models exhibited functional P-glycoprotein and BCRP-mediated efflux; however, organoid models showed more physiological transport behavior, including tighter paracellular barriers and more balanced efflux ratios of the tested reference drugs. While Caco-2 cells displayed exaggerated transporter activity, particularly for BCRP, organoid-derived monolayer models more closely reflected expected drug permeability profiles. Importantly, organoid monolayers retained region-specific gene expression and transporter localization, features absent in the uniform Caco-2 model. Recently, Moyer et al. compared organoid-derived epithelial monolayers generated from jejunal and duodenal organoids with Caco-2 monocultures under both static and fluidic culture conditions. Their study demonstrated that organoid-derived monolayers establish intact epithelial barriers and express functional transporters, and further showed that certain barrier and transport-related readouts were improved under perfused conditions compared to static culture [33]. Our study extends on these two studies by providing a direct side-by-side comparison between organoid-derived monolayers and a Caco-2/HT29 coculture system, and by integrating detailed structural and ultrastructural characterization (SEM, TEM, microvilli organization, mucus distribution), polarized secretome profiling, and barrier function measurements within the same experimental framework. Taken together, these results reinforce the advantages of organoid monolayer cultures as a physiologically relevant system for studying intestinal absorption and drug transport [18].

Overall, our direct comparison of the two intestine models illustrates that both organoid-derived and cell line coculture monolayers successfully form polarized epithelial barriers but differ in key structural and functional aspects. Organoid-derived cultures showed more continuous mucus coverage, lower small-molecule permeability, and physiologically oriented growth factor secretion, whereas the cell line coculture displayed microvilli morphology more closely resembling reported in vivo dimensions, together with higher secretion of angiogenic mediators. These findings underscore that no single model fully recapitulates all aspects of human duodenal physiology; rather, each model provides distinct advantages that should be weighed against the intended context of use.

## Conclusion

This study provides a comprehensive side-by-side comparison of organoid-derived duodenum epithelial monolayers and Caco-2/HT29 cocultures. While both models establish intact barriers and epithelial polarization, organoid cultures more closely reflect homeostatic epithelial signaling and mucus distribution, whereas cell line cocultures better approximate native microvillus length and density. Together, these complementary features highlight the complementary value of both model systems, allowing researchers to prioritize either physiological fidelity or experimental practicality depending on the context. This comparative framework may guide researchers in selecting the most appropriate model for specific contexts, such as drug transport, host-pathogen studies, or epithelial homeostasis.
